# Waning light, waxing pain: The lunar cycle's association with migraine headache occurrence

**DOI:** 10.1111/head.15035

**Published:** 2025-08-28

**Authors:** Alexander Yoo, Brendan Keenan, Angeliki Vgontzas, Murray A. Mittleman, Suzanne M. Bertisch, Ron Anafi

**Affiliations:** ^1^ Department of Medicine, Division of Sleep Medicine University of Pennsylvania Perelman School of Medicine Philadelphia Pennsylvania USA; ^2^ Department of Medicine, Center for Sleep and Circadian Neurobiology Institute for Biomedical Informatics, University of Pennsylvania Perelman School of Medicine Philadelphia Pennsylvania USA; ^3^ Department of Neurology Brigham and Women's Hospital Boston Massachusetts USA; ^4^ Harvard Medical School Boston Massachusetts USA; ^5^ Department of Social and Behavioral Sciences Harvard T.H. Chan School of Public Health Boston Massachusetts USA; ^6^ Cardiovascular Epidemiology Research Unit Beth Israel Deaconess Medical Center Boston Massachusetts USA; ^7^ Division of Sleep and Circadian Disorders Brigham and Women's Hospital Boston Massachusetts USA

**Keywords:** biological rhythms, chronobiology, circalunar rhythms, lunar rhythms, migraine, sleep

## Abstract

**Objective:**

To examine circalunar rhythms in migraine headache occurrence in a prospective cohort of 98 adults with episodic migraine.

**Background:**

Migraine is a prevalent neurological disorder characterized by paroxysmal attacks. While time‐of‐day and seasonal rhythmicity in migraine occurrence have been described, little is known about circalunar patterns. Understanding these rhythms may inform headache prediction and guide personalized preventive medication timing.

**Methods:**

We performed a secondary, post‐hoc analysis using data from a prospective cohort study (March 2016–October 2017). Participants completed twice‐daily electronic diaries, recording various characteristics including headache, and, when applicable, menstrual cycle timing. Participants wore wrist actigraphs for 6 weeks. We tested for a 30‐day circalunar rhythm in headache risk at the population‐level. We then examined for lunar synchronized rhythms in individuals, adjusting our analysis for participant‐specific factors and differences in baseline headache risk. Sleep characteristics and menstrual timing were assessed as potential mediators of the relationship between lunar phase and headache occurrence.

**Results:**

Ninety‐eight participants were followed for a median length of 43 days (interquartile range [IQR]: 42–45 days) with an average of 24.2% (standard deviation [SD] 13.2) of those days being headache days. Population‐level analysis showed a significant relationship between lunar phase and headache risk (trough‐to‐peak odds ratio: 1.2 [95% confidence interval {CI}: 1.04, 1.49]). Individual‐level analysis, adjusted for age, sex/menopausal status, and preventative medication use, showed 1.34 (95% CI: 1.1, 1.72) times higher headache odds at lunar cycle peak versus trough. Risk peaked 1–2 days before new moon. Neither sleep characteristics nor menstrual timing appeared to mediate the lunar phase‐headache relationship.

**Conclusions:**

Headache risk varied with the lunar cycle, with 34% higher odds shortly before new moon compared to before full moon. Sleep and menstrual cycle timing did not appear to explain this relationship, suggesting underlying chronobiological mechanisms. Additional studies are needed to confirm these findings and characterize individual variability in rhythmicity.

**Plain Language Summary:**

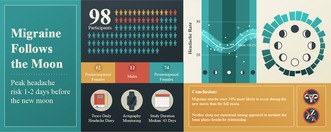

## Plain Language Summary

While migraine headaches can be hard to predict, some patients believe headaches are connected to the moon‘s phases. Our research showed that migraine attacks were 34% more likely to occur during the new moon than during the full moon. These findings may help us understand migraine biology and allow some patients to better time their treatments.

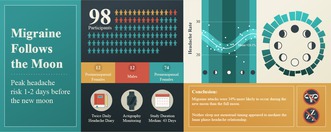

**Abbreviations**
CGRP: calcitonin gene‐related peptide
CI: confidence interval
CSK1D: Casein Kinase 1 delta
d₀: Lunar day 0 (first new moon designation)
DAG: directed Acyclic Graph
GLMM: Generalized Linear Mixed Model
GnRH: gonadotropin‐releasing hormone
ICHD‐3: International Classification of Headache Disorders, 3rd edition
IQR: interquartile range
MESOR: Midline Estimating Statistic of Rhythm
Q1: first quartile
Q3: third quartile
Q‐Q: Quantile‐Quantile (plots)
SD: standard deviation
τ: Tau (lunar cycle period)

## INTRODUCTION

Episodic migraine is a highly prevalent neurological disorder characterized by debilitating paroxysmal headache attacks. The lack of predictability is one of the most challenging aspects of the disease.^1^ Though many patients identify personal triggers,^2^ only a minority can accurately forecast their own attacks.^3^ Predictive models using self‐reported triggers have faced limited success.^4^ The inability to forecast attacks from triggers alone may reflect complex interactions between precipitating and mitigating behaviors and exposures or latent rhythms that influence headache risk outside of these exogenous factors.^5^

Daily rhythms in headache risk are well established and circannual/seasonal rhythms in headache occurrence have also been reported.^6^, ^7^ A meta‐analysis of eight studies showed that 50% of study participants had attacks that occurred most often in the late morning to early evening.^8^ Moreover, a genetic analysis found that approximately two‐thirds of migraine susceptibility genes are circadian clock‐controlled genes.^8^ Seasonal and other rhythmic patterns in migraine occurrence have been proposed, but evidence remains inconclusive due to conflicting findings.^9^

The 29.5‐day lunar cycle is a compelling candidate pattern for variation in headache risk as lunar rhythms have been observed in select behaviors and disorders. Lunar rhythms are observed in sleep duration, with a shorter sleep typically occurring near the full moon.^10^, ^11^, ^12^ In patients with rapidly cycling bipolar disorder, mood fluctuations appear to synchronize with lunar cycles.^13^ Some evidence suggests that intracranial hemorrhage and transient ischemic attacks may occur more frequently during certain moon phases.^14^ Indeed, approximate 30‐day cycles in migraine risk have already been appreciated and have most commonly been ascribed to monthly estrogen withdrawal since the 1970s.^15^ These “menstrual migraine attacks” may occur so predictably that they can be anticipated and preemptively treated with long‐acting medication.^16^ However, recent critical analysis reveals that the evidence supporting this long‐standing hypothesis remains surprisingly limited and inconsistent, raising the possibility that these monthly migraine patterns, like other biological processes, might be related to the moon.^17^, ^18^ In fact, a recent retrospective study, although constrained by the coarse temporal resolution of their data, provided some suggestive evidence of lunar rhythms in migraine in children.^19^

Identifying a circalunar component to migraine rhythmicity would likely improve our ability to predict attacks and could point to previously underexplored biological factors influencing migraine occurrence. However, evidence for lunar influences on migraine risk remains lacking, as no studies have evaluated this relationship in a well‐characterized cohort. We tested the hypothesis that headache risk varies systematically across the 29.5‐day synodic lunar cycle in a prospective cohort of adults with episodic migraine.

## METHODS

### Study setting and overview

This secondary, post‐hoc analysis used data from a prospective cohort study originally examining associations between nightly sleep and migraine.^20^ Participants with episodic migraine, per the International Classification of Headache Disorders‐3 (ICHD‐3), were recruited from three Boston hospitals and local colleges between March 2016 and October 2017. Of 126 interested individuals, 98 met eligibility criteria and provided sufficient data (at least 21 days). Three eligible individuals withdrew <3 weeks into the observation period. Participants completed baseline surveys on sociodemographics, medical history, psychosocial, and lifestyle factors. Over the 6‐week study period, participants completed twice‐daily diaries documenting headaches and, where applicable, menstrual cycle timing. The original study was approved by Beth Israel Deaconess Medical Center's Committee on Clinical Investigations, and all participants provided written informed consent. The present study was granted exemption from review by the Institutional Review Board of the University of Pennsylvania (Protocol number 853601).

### Study sample

The cohort included individuals aged 18 or older meeting ICHD‐3 criteria for episodic migraine, with or without aura. Diagnoses were confirmed by physician interviews. Participation required a minimum 3‐year history of migraine with 2–14 monthly migraine episodes over the past 3 months and the ability to communicate in English. Exclusion criteria were other chronic pain conditions, untreated obstructive sleep apnea (known or high‐risk per Berlin Questionnaire^21^), current opioid use, pregnancy, unmanaged medical conditions interfering with participation, or completion of fewer than four out of seven daily diary entries during screening. No power calculation was conducted prior to the present study. The sample size for this secondary analysis was based on available data.

### Assessments

#### Headaches

The primary outcome was the presence of self‐reported headaches on any given calendar day. Participants completed electronic diaries morning and evening, recording any headache occurrence. For each headache, they documented start and end times. Calendar days were flagged as “headache days” if they coincided with a headache start date, an end date, or days between these two points.

#### Lunar phase

We assigned synodic lunar phases using new moon dates from the Astronomical Applications Department of the United States Naval Observatory.^22^ The first new moon of each participant's collection period was designated as lunar day 0 (denoted as d_0_). Given our daily temporal resolution and the 29.5‐day period of the synodic lunar cycle, we mapped calendar days to a 30‐day lunar cycle (represented as τ) and converted lunar phase to radians, as demonstrated symbolically below. We repeated this analysis assuming a 29‐day cycle to test our findings' robustness.

$$\theta =\left[\left(d-d_{0}\right)\;\mathrm{mod}\;\tau \right]\cdot \frac{2\pi }{\tau }$$

#### Covariates

We collected baseline characteristics such as sex, age, and reproductive stage. Sleep measures were derived from actigraphic data (Actiwatch Spectrum; Philips Respironics, Murraysville, PA). Participants were instructed to wear devices continuously on the non‐dominant wrist for 42 days. The device captured physical activity in 30‐second epochs and off‐wrist time. Actigraphy data were analyzed at the Brigham and Women's Health Sleep Reading Center by a certified technician blinded to daily headache data. A hierarchical approach determined each rest period's start and stop time.^23^ The Actiware 6.0 algorithm was then used to classify each 30‐second epoch of annotated rest periods as either sleep or wake. Sleep duration and efficiency were calculated from this data.

A priori, we identified demographic and clinical variables (sex, menopausal status, preventative medication use, age), that might influence migraine risk and improve the precision of our lunar phase effect estimates. Separately we identified potential mediators (sleep duration, sleep efficiency, and menstrual phase) for the lunar phase‐headache relationship. To account for both sex and menopausal status, we created a composite categorical variable (referred to as “sex/menopausal status”) stratifying participants into male sex, premenopausal females, and post‐menopausal females. Females were classified as pre‐ or post‐menopausal based on self‐report. Those unsure and under 52 (median U.S. menopause age^24^) were deemed premenopausal (*n* = 2). Females uncertain and ≥52 years old were classified as post‐menopausal (*n* = 1).

For premenopausal females who recorded menstrual bleeding, we defined their cycle length as the span between the first reported menstrual bleeding day and the next subsequent bleeding day ≥20 days later. For participants with insufficient data (only one cluster of bleeding days), we assumed a 28‐day cycle, the median length for females in their third decade.^25^

Migraine prophylaxis medication use was represented as a binary variable.

We removed each participant's first day of data since the previous night's sleep information was unavailable. For the remaining observations, approximately 8.2% lacked actigraphy sleep data. We addressed these missing values using multiple imputation by chained equations, generating five imputed datasets with 50 iterations each.^26^ The imputation model incorporated behavioral factors (such as prior day caffeine/alcohol intake and physical activity), participant characteristics, and time‐varying covariates while accounting for within‐participant clustering. Convergence was verified through trace plots showing adequate mixing and Rhat values between 1 and 1.01.

### Statistical analysis

Continuous variables were assessed for normality using Shapiro–Wilk tests, visual inspection of histograms, and Q‐Q plots. Normally distributed data are presented as mean ± standard deviation, while non‐normal variables are reported as median [interquartile range]. Categorical variables are shown as frequencies and percentages.

We employed two complementary approaches to assess the circalunar periodicity of migraine headache occurrence: aggregated, population‐level analysis and individual‐level, daily data analysis. The aggregate analysis offers a visually interpretable representation of cyclical patterns in headache occurrence. The individual‐level analysis provides a more robust statistical framework by incorporating participant‐specific factors.

Prior to our main analyses, we tested whether the statistical assumptions of our models were met by the data. For the population‐level analysis, we examined the mean–variance relationship of aggregated headache counts. We found modest under‐dispersion, leading us to employ a generalized Poisson model. For individual‐level analysis using generalized linear mixed models (GLMMs), including those used in mediation analyses, we verified assumptions through Q‐Q and residual plots, testing for distribution appropriateness, dispersion, and outliers. Diagnostic tests confirmed these assumptions were met, except for our sleep efficiency variable which is discussed below.

We first used Rayleigh's test to assess whether headache events clustered around a single peak within the lunar cycle or were uniformly distributed throughout. We then employed cosinor regression^27^ to model the relationship between lunar phase and the aggregated rate of headache attacks. Data were modeled using a generalized Poisson distribution accounting for the relative under‐dispersion. The model included the sine and cosine components of lunar phase as independent variables:

$$\log\left(E\left(Y_{d}\right)\right)=\beta_{0}+\beta_{1}\cos\left(\frac{2\pi d}{\tau }\right)+\beta_{2}\sin\left(\frac{2\pi d}{\tau }\right)+\log\left(N_{d}\right)$$

where *E*(*Y*_*d*_) is the expected headache count on lunar‐day *d*, *τ* is the 30‐day lunar period, and *N*_*d*_ is the offset term representing the number of observed person‐days of at a given lunar phase. The significance of the lunar phase terms was assessed using an *F*‐test. We estimated the difference between peak and trough headache chance (twice the log‐odds amplitude, exponentiated to obtain the odds ratios), the timing of the peak (acrophase), and the average chance of headache across the lunar cycle (midline estimating statistic of rhythm, “MESOR”). The estimated population rate of headache occurrence on a given lunar day is computed as *E*(*Y*_*d*_)/(*N*_*d*_).

For the individual‐level analysis, we used GLMMs to estimate the relationship of lunar phase with daily headache odds while accounting for participant‐specific factors. Our base GLMM included fixed effects of age, sex/menopausal status, preventative medication use, and a random intercept term to account for repeated measures within each participant. We then added the sine and cosine of lunar phase to the base model and evaluated the significance of the lunar phase terms using a likelihood ratio test. Amplitude and acrophase were computed from model coefficients. The MESOR was obtained from the model's intercept and represents baseline headache risk when averaged across the lunar cycle. The 95% confidence intervals were determined by bootstrapping with 1000 resamples and taking the 2.5th and 97.5th percentiles as the confidence bounds.^28^

We repeated the above steps using a 29‐day lunar cycle to assess the robustness of our results.

Several studies have suggested that sleep and menstrual timing may influence migraine risk and exhibit circalunar rhythmicity.^12^, ^15^, ^29^, ^30^ We performed exploratory mediation analyses^31^ to investigate if sleep parameters or menstrual cycle timing appeared to underlie the relationship between lunar phase and headache risk. As detailed above, the influence of lunar phase on headache risk was modeled using sine and cosine terms to capture cyclicity. As the individual sine and cosine coefficients are not easily interpretable, we instead calculated amplitudes using these coefficients (amplitude = √(β_sin^2^ + β_cos^2^)) to examine the direct and indirect effects of lunar phase. The total influence of lunar rhythms was partitioned into direct effects attributable to lunar phase and indirect effects reflecting the influence of the lunar phase acting through potential mediating pathways. Analyses were performed separately for each of the mediators (sleep duration, sleep efficiency, timing from menstrual onset) using the product of coefficients method^32^ to estimate the indirect effect. We calculated 95% bootstrap confidence intervals based on 1000 bootstrap resamples. Sleep efficiency showed outlier values in diagnostic tests. We repeated this analysis after a logit transformation of this potential mediator. This transformation did not substantively alter the partitioning of direct and indirect lunar effects.

All hypothesis tests were two‐tailed and considered significant at a *p*‐value less than 0.05.

All analyses were performed using R version 4.3.1 (R Foundation for Statistical Computing, Vienna, Austria; www.r‐project.org) and were conducted with the following libraries: mice^33^ (v3.17.0), circular^34^ (v0.5–1), DHARMa^35^ (v0.4.7), lme4^36^ (v1.1–36), glmmTMB^37^ (v1.1.10), and mediation^38^ (v4.5.0).

## RESULTS

### Participant characteristics and data summary

Characteristics of the study participants are provided in Table 1. Participants were followed for a median of 43 [IQR: 42–45] days, about 1.5 lunar cycles. There was a total of 4308 observation days. Premenopausal females (*n* = 74) experienced headaches on an average of 25.3% (standard deviation [SD]: 12%) of the recorded days. Postmenopausal females (*n* = 12) averaged 30.7% (SD: 14.1%), while males (*n* = 12) averaged 9.6% (SD: 7.2%).

**TABLE 1:** Distribution of headache days and average sleep characteristics by sex and menopausal status.

Characteristic	All	Pre‐menopausal women	Post‐menopausal women	Men
Total participants, *n* (%)	98	74 (75.5)	12 (12.2)	12 (12.2)
Per subject observation length, median [Q1–Q3]	43 [42–45]	43 [42–45]	44 [43–47]	42 [41–49]^a^
No. of participants with migraine with aura, *n* (%)	32 (32.7)	21 (28.4)	7 (58.3)	4 (33.3)
Age, years, median [Q1–Q3]	32 [25–41]	31 [25–39]	53 [52–56]	31 [23–39]
Percentage of headache days per participant, mean (SD)	24.2 (13.2)	25.3 (12)	30.7 (14.1)	9.6 (7.2)
HIT‐6 score, mean [Q1–Q3]^b^	61 [57–65]	61 [58–65]	64 [61–67]	59 [52–63]
No. of participants on preventative medication, *n* (%)	26 (26.5)	19 (25.7)	6 (50)	1 (8.3)
Sleep efficiency on prior night, mean (SD)	89.5 (3.1)	89.3 (4.7)	90.4 (3.8)	89.5 (4.8)
Sleep duration prior night, hours, mean (SD)	7.3 (0.6)	7.3 (1.4)	7.3 (1.3)	6.9 (1.2)
Insomnia Severity Index score, median [Q1–Q3]^c^	7 [3–11]	7 [3–12]	3 [2–11]	5 [3–9]
Center for Epidemiologic Studies Depression Scale‐20 score, median [Q1–Q3]^d^	7.5 [4–14]	8 [4–14]	7 [5–15]	7 [5–12]

^a^ For male participants, length of observation is reported as median [range] due to the high frequency of identical values at the quartiles, while for other participants, the standard median [Q1–Q3] format is used.

^b^ Scores ≥60 indicate severe impact of headache on the patient's life; ≥56 indicates substantial impact.

^c^ Scores ≥8 indicate subthreshold insomnia, ≥15 indicate moderate or more severe clinical insomnia.

^d^ Scores ≥16 indicate risk for clinical depression.

### Relationship between lunar cycle and headache occurrence

**FIGURE 1:**
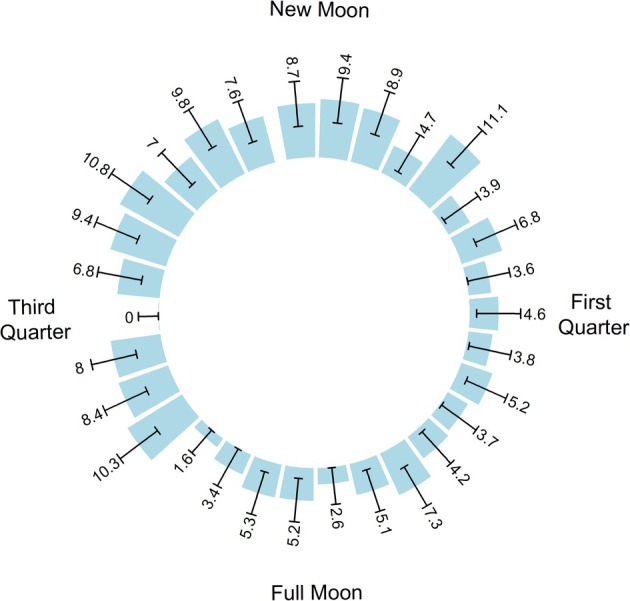
Additional headaches per 100 days as a function of lunar phase. This rose plot depicts the relative aggregate number of headaches across all participants per each day in an assumed 30‐day lunar period arranged clockwise. The numbers associated with each bar represents additional headaches per 100 days above the minimum per 100 days (20 headaches, Third Quarter). Error bars represent ±1 standard error of proportion of headache‐days and are calculated using the formula for the standard error of the mean for a generalized Poisson distribution. The plot depicts a clustering of headaches tending to occur prior to the new moon and a nadir in headaches around the first quarter and full moon. [Color figure can be viewed at wileyonlinelibrary.com]

Analyzing the entire study population, Rayleigh's test of uniformity demonstrated a non‐uniform distribution of headaches across the lunar cycle (*p* = 0.042). Headache occurrences clustered around 1.2 days prior to the new moon. Figure 1 illustrates the distribution of headache rates relative to the lunar cycle quarters.

**FIGURE 2:**
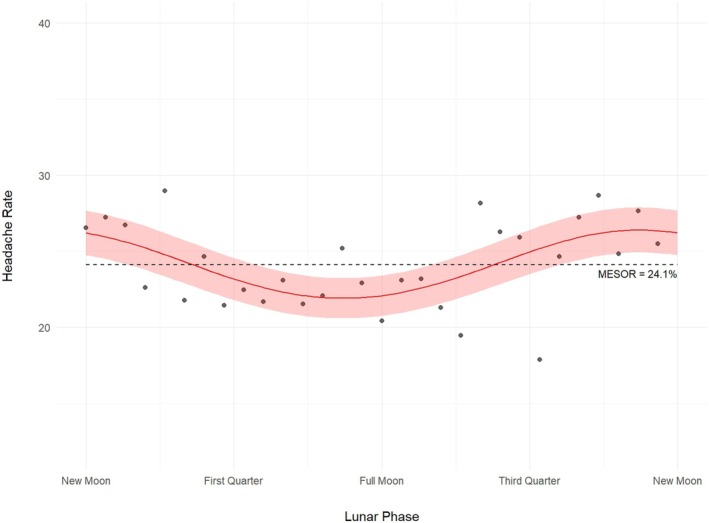
Population‐level risk of headache occurrence vs. lunar phase. Cosinor regression lines plotted with actual headache risk across the lunar cycle. The analysis employed generalized Poisson regression to model the count of headaches using the number of lunar days observed as the offset. Lunar phase is significant (*p* = 0.003) in a model including all participants. The maximum difference from the average rate (i.e., the amplitude) is 1.1 times the average risk. Headache risk peaked 1.9 days before the new moon. The 95% confidence interval in headache risk is represented by the light red band. [Color figure can be viewed at wileyonlinelibrary.com]

Similarly, a cosinor model of aggregate headache rate in our study sample showed circalunar rhythmicity (*p* = 0.003). The average headache rate over a complete lunar cycle (MESOR) was 24.1% (95% confidence interval [CI]: 21.6, 26.5%). The peak and trough rates during the lunar cycle were 26.3% and 22.1% (amplitude [95% CI] of 1.1 [1.05, 1.39] times higher than the average rate), respectively. Acrophase, or the timing of peak headache rate, was approximately 1.94 days (95% CI: −7.68, 4.70 days) before the new moon. The fitted cosinor model and headache rate data are shown in Figure 2.

Our individual‐level GLMM adjusting for age, sex/menopausal status, and preventative medication use demonstrated a relationship between lunar phase and the odds of headache occurrence (*p* = 0.023). The peak odds of headache occurred 1.61 days before the new moon (95% CI: −5.87, 3.15 days). The peak odds of headache were 1.16 times higher (95% CI: 1.05, 1.31) than the MESOR and 1.34 (95% CI: 1.1, 1.72) times higher than trough risk. These results align with our population‐level findings. The baseline odds of headache occurrence averaged across the lunar cycle (i.e., the MESOR) was 0.28 (95% CI: 0.24, 0.32) for premenopausal females, 0.39 (95% CI: 0.26, 0.54) for postmenopausal females, and 0.09 (95% CI: 0.06, 0.16) for males.

Results did not substantively differ in supplemental analyses assuming a 29‐day, rather than a 30‐day, lunar cycle (see Table S1).

### Mediation analysis

**FIGURE 3:**
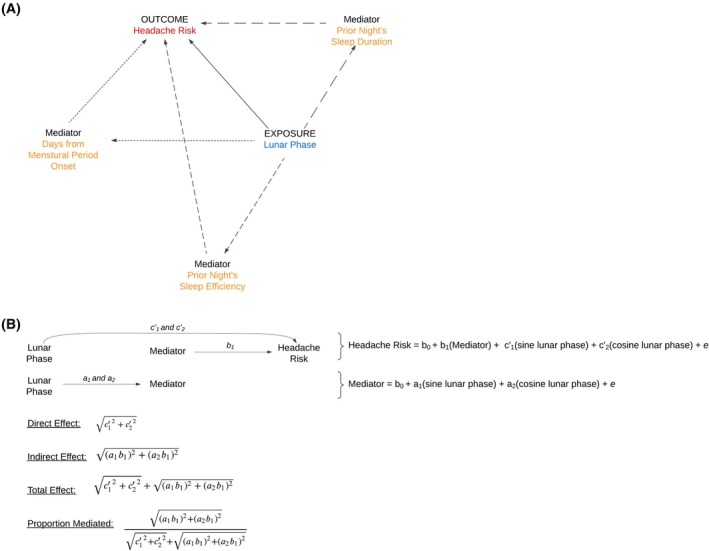
Directed acyclic graph (DAG) of relationships between lunar phase, mediators, and headache risk. (A) The DAG illustrates the hypothesized relationships between the exposure (lunar phase), the mediators (prior night's sleep efficiency, prior night's sleep duration, and days from menstrual period onset), and the outcome (headache risk). Arrows represent the direction of hypothesized causal effects. (B) Corresponding regression equations and effect calculations. The labels on the arrows in the DAG correspond to the regression coefficients in the equations. The a_1_ and a_2_ represent the coefficients for the effect of the sine and cosine of the lunar phase on the mediator, respectively. b_1_ represents the coefficient for the effect of the mediator on headache risk. The c’_1_ and c’_2_ represent the coefficients for the direct effect of the sine and cosine of the lunar phase on headache risk. The equations show the regression models used to estimate these direct and indirect effects. The “Direct Effect” shows the combined effect of the sine and cosine lunar phase terms directly on headache risk. The “Indirect Effect” shows the combined effect of the sine and cosine lunar phase terms on headache risk through the mediator. The “Total Effect” is the sum of the direct and indirect effects. The “Proportion Mediated” represents the proportion of the total effect that is explained by the mediator. In the equations, B_0_ represents the intercept, and ‘e’ represents the error term. Covariates are included in both models but are not depicted in the DAGs for simplicity. [Color figure can be viewed at wileyonlinelibrary.com]

Our analysis (Figure 3) suggested the association between lunar phases and headache risk was not meaningfully explained by the mediating factors we explored (Table 2). Sleep characteristics (efficiency and duration) provided little to no evidence of mediation (proportion of total effects mediated: indirect/total effects <1%). Menstrual period timing showed a larger estimated mediating effect, with this variable accounting for approximately 8% of the total lunar effect (95% CI: 0%–17%).

**TABLE 2:** Causal mediation results.

Metric	Mediators		
	Sleep efficiency	Sleep duration	Days from menstrual period onset
Direct effect^a^, ^b^	1.16 (1.05, 1.33)	1.15 (1.03, 1.33)	1.30 (1.10, 1.62)
Indirect effect^a^, ^c^	1.000 (1.000, 1.002)	1.000 (1.000, 1.002)	1.02 (1.00, 1.04)
Total effect^a^, ^d^	1.16 (1.05, 1.33)	1.15 (1.03, 1.33)	1.30 (1.11, 1.66)
Proportion mediated^e^	0 (0, 0.02)	0 (0, 0.02)	0.08 (0, 0.17)

^a^ Direct, indirect, and total effects are shown as odds ratios and represent the amplitude of lunar phase.

^b^ Direct effects are calculated from the coefficients in the fully adjusted outcome model as √(c_1_^2^ + c_2_^2^): headache risk ~ b_1_(mediator) + c_1_(sine lunar phase) + c_2_(cosine lunar phase) + covariates.

^c^ Indirect effects are computed using the product of coefficients method. Specifically, the coefficients of the mediator in the outcome model are multiplied by the corresponding coefficients of the lunar phase terms (sine and cosine) from the mediator model. This is represented by the formula: √((a_1_b_1_)^2^ + (a_2_b_2_)^2^), where ‘a’ represents coefficients from the mediator model and ‘b’ represents coefficients from the outcome model. The mediator model is represented by the regression equation: mediator ~ a_1_(sine lunar phase) + a_2_(cosine lunar phase) + covariates. The outcome model is represented as: headache risk ~ b_1_(mediator) + c_1_(sine lunar phase) + c_2_(cosine lunar phase) + covariates.

^d^ Total effects is the exponentiated value of the sum of the direct and indirect effects in log odds.

^e^ Proportion mediated is the ratio: indirect effects/total effects.

## DISCUSSION

In this post‐hoc, secondary analysis of a prospective cohort study, we tracked headache occurrence, sleep metrics, and menstrual cycle data for 98 participants meeting International Classification of Headache Disorders 3 criteria for episodic migraine, with or without aura, over an approximate 6‐week duration. Our analysis revealed an association between lunar phase and headache risk in both population‐level of aggregated data and in individual‐level models that accounted for differences in baseline risk along with demographic, reproductive, and preventative medication use information. Headache odds peaked 1.6 days before the new moon and was 1.34 times higher than the odds of headache near the full moon. Exploratory mediation analysis showed that neither sleep nor menstrual cycle phase appear to be dominant factors underlying the relationship between migraine occurrence and lunar phase.

To our knowledge, this is the first prospective characterization of circalunar periodicity in headache in adults with episodic migraine. A prior retrospective study in children similarly found evidence of circalunar migraine attacks.^19^ Consistent with our study, the authors found headaches occurring most frequently during the new moon and third quarter and least commonly during the full moon.

While circalunar rhythms remain relatively understudied in humans, they are widely observed throughout nature and appear to be evolutionarily conserved across diverse species.^39^ Lunar‐synchronized biological rhythms are observed in various marine species, including reproductive cycles in corals and fish, melatonin variation in rabbit fish, melatonin receptor expression in grass puffers, and GnRH‐like hormone expression in marine bristle worms.^40^

How might lunar cycles “talk to the body” and affect headache physiology and migraine risk? Two broad and potentially complementary mechanisms are possible. The first involves direct physiological responses to lunar‐driven environmental changes. Variation in moon light is the most prominent manifestation of the lunar cycle. Yet, light exposure has not been clearly associated with migraine.^41^ Moreover, in an urban environment like the Boston metropolitan area, where our sample resides, the intensity of ambient artificial lighting overwhelms moonlight^42^ making it unlikely that lunar light played a marked role in our results. Low atmospheric pressure may trigger headaches in susceptible individuals. While barometric pressure varies with the lunar cycle,^43^ these changes are below the threshold thought to provoke migraine.^44^ Research in mice has implicated gravitational forces in producing subtle morphological changes in melatonin producing pinealocytes throughout the lunar cycle.^45^ Melatonin may modulate migraine pathophysiology: it possesses antinociceptive properties;^46^ patients with migraine exhibit reduced nocturnal melatonin levels compared with healthy controls,^47^ and supplemental melatonin has shown potential efficacy in both acute and prophylactic migraine treatment.^47^, ^48^ However, this “direct‐to‐headache” mechanism seems less likely as the role of melatonin in migraine pathophysiology remains uncertain and changes in melatonin levels observed across lunar phases are relatively small^12^ compared to day‐night differences, which may be 10–50× greater.

The second mechanism involves *endogenous* molecular or hormonal rhythms that synchronize to lunar phase, similar to how our internal circadian clock entrains to the day‐night cycle.^49^ Endogenous circalunar rhythms are well‐documented across marine species, with organisms like the bristle worm *Platynereis dumerilii* maintaining precise monthly reproductive cycles even in laboratory settings without lunar cues.^40^ Molecular studies show that lunar phases influence circadian clock gene expression in free‐running conditions, demonstrating sophisticated internal timing mechanisms.^40^ These biological models provide a plausible framework for how circalunar cycles might influence migraine pathophysiology through internal oscillators. Indeed, while lunar driven changes in melatonin synthesis might be too subtle to directly modulate headache risk, they might still act to entrain vestigial, endogenous oscillators or otherwise alter neuroendocrine signaling. The subtle lunar rhythms observed in melatonin levels might also be present in other, yet unstudied, neuroendocrine parameters. Moving forward, techniques like subcutaneous microdialysis^50^ may allow for a more comprehensive picture of longer‐term rhythms across various physiological systems. While any single lunar‐driven change might be individually subtle, the collective effects of direct lunar outputs or lunar‐entrained outputs might exert a meaningful influence on migraine attack susceptibility.

An intriguing candidate to connect headache pathophysiology and circalunar clocks is Casein Kinase 1 delta (CSK1D). CSK1D is an evolutionarily conserved circadian clock modulator linked to familial migraine.^51^, ^52^ In the marine crustacean Eurydice pulchra, CSK1D has been implicated in circatidal rhythm regulation.^53^ In humans, a recent study provided suggestive evidence that CSK1D exhibits both ~12 and ~ 24 hour rhythmicity.^54^ The interaction of circatidal (12.4‐hour) and circadian (24‐hour) rhythms would theoretically generate a “beat” pattern with an approximate 30‐day period in this headache‐associated gene.^55^ Plausibly, this beat pattern could entrain to lunar cues leading to synchronization with lunar rhythms, similar to the monthly differential gene expression patterns seen in *P. dumerilii*.

Regardless of mechanism, circalunar rhythms in headache could inform treatment timing. Chronotherapy aims to optimally time treatment to enhance therapeutic benefits and minimize adverse effects.^56^ While the focus of chronotherapy has largely been on circadian rhythms, these principles can also be applied to rhythms of other lengths. Proper timing of calcitonin gene‐related peptide (CGRP) ‐monoclonal antibody therapy may be impactful in individuals with a pronounced circalunar headache rhythm where the differences in risk may be clinically meaningful. The 28‐day terminal half‐lives of erenumab and galcanezumab^57^, ^58^ make it possible to misalign dosing such that the concentration of the drug reaches its nadir the same time headaches are most likely to occur. This circumstance could exacerbate perceived “wearing off effects,”^59^ or the belief that the treatment is ineffective. Conversely, precisely timed dosing might more effectively suppress potential breakthrough headaches.

Timing headache treatments to lunar cycles carries minimal risks while potentially benefitting patients with clinically leverageable circalunar risk patterns. We did not have sufficient data to assess interindividual variations in circalunar headache patterns. Since time‐of‐day variation in headache is not universal,^8^ circalunar periodicity may also only be present in a subpopulation of patients with migraine. Detecting individual rhythms for clinical benefit would require extended monitoring and automated decision support tools. Mobile applications which facilitate headache documentation already enhance patients' ability to capture headaches over the longer term. In the future, passive wearable technologies might be able to detect migraine attacks through a combination of autonomic signals, activity, and ambient light, further enhancing longitudinal data collection over an extended time horizon.

One of the key limitations of our study is its restricted generalizability. Our results may not generalize to older populations, those with different migraine profiles (i.e., chronic migraine), or individuals from different geographical regions. While the more general effect of lunar light is uncertain, our study was conducted in the Boston metropolitan area where urban light pollution likely attenuated any lunar illumination effects. Also, warmer, less urban environments might result in greater lunar light exposure as more time is spent outside. Lunar gravitational effects may vary according to latitude and therefore could theoretically exert geographically dependent effects on headache risk. Future studies should examine these relationships in diverse geographic settings to better understand how environmental contexts might influence lunar‐migraine associations. Additionally, while we rigorously captured over 4000 days of headache data over a 6‐week period, this window was insufficient for examining intraindividual‐level circalunar patterns, which would likely require observation of several moon cycles within individuals. Data from individual‐level analyses could also clarify whether the observed rhythms differ across sexes and menstrual status, or if they even exist in all groups. Finally, while we conducted exploratory mediation analyses for sleep and menstrual timing, we did not evaluate other potential mediators or coincidental factors including weather or light exposure that might vary with lunar phase in our study. The inclusion of these covariates could, in theory, refine our estimate of the lunar effect on headache risk. Nonetheless given their controversial influence on migraine attack susceptibility,^41^, ^60^ their omission likely represents a minor limitation in our modeling approach.

Our study has several strengths. Whereas prior studies evaluating relationships between lunar phase and health outcomes have relied on data with temporal resolution limited to the four principle lunar phases, our daily‐level analysis enabled a more precise assessment of lunar phase and its association with headache risk. Additionally, the concurrent collection of sleep characteristics and menstrual timing data allowed us to evaluate potential mediators, helping determine whether the lunar phase association was independent of or partially mediated by these processes. Finally, our multiple statistical approaches with concordant results strengthen confidence in the findings.

In conclusion, in this prospective cohort of otherwise healthy adults with episodic migraine, we observed an association between lunar phase and headache risk, with the odds of headache being 34% greater at the peak compared to the trough. Peak risk occurred shortly before the new moon. This effect was not substantially explained by changes in prior night's sleep or menstrual cycle timing. Additional studies are needed to confirm this association in more diverse populations and characterize interindividual differences in migraine rhythmicity. In addition to suggesting new directions in investigating migraine pathophysiology, identification of circalunar rhythms in certain individuals may provide practical utility in optimizing timing of certain preventative treatments.

## AUTHOR CONTRIBUTIONS

**Alexander Yoo:** Conceptualization; data curation; formal analysis; investigation; methodology; resources; software; supervision; validation; visualization; writing – original draft; writing – review and editing. **Brendan Keenan:** Formal analysis; methodology; validation; writing – review and editing. **Angeliki Vgontzas:** Formal analysis; methodology; writing – review and editing. **Murray A. Mittleman:** Formal analysis; methodology; writing – review and editing. **Suzanne M. Bertisch:** Data curation; formal analysis; funding acquisition; methodology; writing – review and editing. **Ron Anafi:** Conceptualization; formal analysis; methodology; supervision; validation; writing – original draft; writing – review and editing.

## FUNDING INFORMATION

No industry sponsored this work. This work was funded by grants from the National Institutes of Health (T32HL007953), National Institute of Neurologic Disorders and Stroke (R21‐NS091627) and the American Sleep Medicine Foundation. This work was also conducted with support from Harvard Catalyst | The Harvard Clinical and Translational Science Center (National Center for Advancing Translational Sciences, NIH Award UL 1TR002541) and financial contributions from Harvard University and its affiliated academic health care centers.

## CONFLICT OF INTEREST STATEMENT

**Suzanne M. Bertisch** has received consultant fees from Idorsia and Apnimed. **Alexander Yoo, Brendan Keenan, Angeliki Vgontzas, Murray A. Mittleman**, and **Ron Anafi** declare no conflicts of interest.

## Supporting information


Data S1.
