# Supplementary material for: Waning light, waxing pain: The lunar cycle's association with migraine headache occurrence
Source: Headache. 2025 Aug 28;66(3):584–94. doi: 10.1111/head.15035 (PMC12951705; doi:10.1111/head.15035)
Supplement: Supplementary file 1 — Data S1. [file HEAD-66-584-s001.docx]

Supplemental Table 1: Influence of 29 and 30-Day Lunar Cycles on Headache Risk, Population and Daily-Level Models

|  |  | **Rhythm Statistics** | | | |
| --- | --- | --- | --- | --- | --- |
| Cycle*^1^* | F-Test P-value*^2^* | Amplitude (95% CI)*^3,4^* | MESOR (95% CI)*^5,4^* | Trough to Peak | Acrophase (95% CI)*^6,4^* |
| Population-Level | | | | | |
| 29-Day Cycle | 0.006 | 1.10 (1.02, 1.2) | 0.24 (0.21, 0.27) | 1.21 (1.04, 1.44) | -1.53 (-6.69, 5.07) |
| 30-Day Cycle | 0.003 | 1.10 (1.02, 1.22) | 0.24 (0.22, 0.26) | 1.21 (1.04, 1.49) | -1.94 (-7.68, 4.70) |
| Daily-Level | | | | | |
| 29-Day Cycle | 0.028 | 1.15 (1.05, 1.33) | 0.28 (0.24, 0.32) | 1.33 (1.1, 1.77) | -1.17 (-5.12, 4.52) |
| 30-Day Cycle | 0.023 | 1.16 (1.05, 1.31) | 0.28 (0.24, 0.32) | 1.34 (1.1, 1.72) | -1.61 (-5.87, 3.15) |
| *^1^*Adjusted for age. | | | | | |
| *^2^*F-tests calculated p-values. The daily-level analysis compared a model including lunar phase, age and sex/menopausal status to one only containing age and sex/menopausal status. These tests assessed whether lunar phase terms significantly improved model fit. | | | | | |
| *^3^*Represented as odds ratios in daily-level models. | | | | | |
| *^4^*Bootstrapping was utilized to obtain a distribution of each rhythm statistic and the 2.5% and 97.5% quantiles were used as bounds for the 95% confidence interval. | | | | | |
| *^5^*Represented as odds. | | | | | |
| *^6^*Presented as days before or after the new moon. A negative value indicates number of days in the counter-clockwise (movement towards the third quarter) direction. | | | | | |
